# Innovation and Emerging Roles of *Populus trichocarpa* TEOSINTE BRANCHED1/CYCLOIDEA/PROLIFERATING CELL FACTOR Transcription Factors in Abiotic Stresses by Whole-Genome Duplication

**DOI:** 10.3389/fpls.2022.850064

**Published:** 2022-03-09

**Authors:** Shuo Wang, Yirong Shen, Liangyu Guo, Lingling Tan, Xiaoxue Ye, Yanmei Yang, Xijuan Zhao, Yuqi Nie, Deyin Deng, Shenkui Liu, Wenwu Wu

**Affiliations:** ^1^State Key Laboratory of Subtropical Silviculture, Zhejiang Agriculture and Forestry University, Hangzhou, China; ^2^Institute of Tropical Bioscience and Biotechnology, Chinese Academy of Tropical Agricultural Sciences, Haikou, China

**Keywords:** TCP, whole-genome duplication, K-Pg extinction event, abiotic stress, adaption, transcriptomics

## Abstract

The TEOSINTE BRANCHED1/CYCLOIDEA/PROLIFERATING CELL FACTOR (TCP) family proteins are plant-specific transcription factors that have been well-acknowledged for designing the architectures of plant branch, shoot, and inflorescence. However, evidence for their innovation and emerging role in abiotic stress has been lacking. In this study, we identified a total of 36 *TCP* genes in *Populus trichocarpa*, 50% more than that in *Arabidopsis* (i.e., 24). Comparative intra-genomes showed that such significant innovation was mainly due to the most recent whole genome duplication (rWGD) in *Populus* lineage around Cretaceous-Paleogene (K-Pg) boundary after the divergence from *Arabidopsis*. Transcriptome analysis showed that the expressions of *PtrTCP* genes varied among leaf, stem, and root, and they could also be elaborately regulated by abiotic stresses (e.g., cold and salt). Moreover, co-expression network identified a cold-associated regulatory module including *PtrTCP31*, *PtrTCP10*, and *PtrTCP36*. Of them, *PtrTCP10* was rWGD-duplicated from *PtrTCP31* and evolved a strong capability of cold induction, which might suggest a neofunctionalization of *PtrTCP* genes and contribute to the adaptation of *Populus* lineage during the Cenozoic global cooling. Evidentially, overexpression of *PtrTCP10* into *Arabidopsis* increased freezing tolerance and salt susceptibility. Integrating co-expression network and *cis-*regulatory element analysis confirmed that PtrTCP10 can regulate the well-known cold- and salt-relevant genes (e.g., *ZAT10*, *GolS2*, and *SOS1*), proving that PtrTCP10 is an evolutionary innovation in *P. trichocarpa* response to environmental changes. Altogether, our results provide evidence of the rWGD in *P. trichocarpa* responsible for the innovation of *PtrTCP* genes and their emerging roles in environmental stresses.

## Introduction

Plant development and defense to various environmental stresses are determined by the coordinate transcriptional regulation of multiple genes. This is mostly achieved by the action of transcription factors, which show specific DNA binding to regulate downstream genes in response to endogenous and exogenous stimuli (Riechmann et al., 2000). TEOSINTE BRANCHED1/CYCLOIDEA/PROLIFERATING CELL FACTOR (TCP) proteins constitute a plant-specific transcription factor family that plays important roles in multiple developmental processes of plants (Danisman et al., 2013). The TCP family is named after the initially identified members, including *TEOSINTE BRANCHED1* (*TB1*) in maize (*Zea mays*), *CYCLOIDEA* (*CYC*) in snapdragon (*Antirrhinum majus*), and *PCF* genes in rice (*Oryza sativa*) (Luo et al., 1996; Doebley et al., 1997; Kosugi and Ohashi, 1997; Cubas et al., 1999), which share a 59-residue conserved non-canonical basic helix-loop-helix (bHLH) DNA-binding region (i.e., TCP domain) (Cubas et al., 1999). Based on the domain, TCP family proteins can be classified into two subfamilies, including class I (TCP-P) and class II (TCP-C), and the class II subfamily can be further subdivided into two lineages, namely, ubiquitous CINCINNATA (CIN) and angiosperm-specific CYC/TB1 (Kosugi and Ohashi, 2002; Navaud et al., 2007; Li, 2015). In addition to the TCP domain, several class II members also have an R-domain, which is predicted to form a hydrophilic α-helix or coiled coil structure involved in protein-protein interactions (Lupas et al., 1991).

The basic region of TCP domain is essential for recognizing GC-rich *cis*-regulatory elements (Kosugi and Ohashi, 2002; Li et al., 2005; Viola et al., 2011; Danisman, 2016). Random binding site selection experiments suggested that class I proteins can recognize the consensus DNA sequence GTGGGNCC, whereas class II proteins show a preference binding for GTGGNCCC in *Arabidopsis* and rice (Kosugi and Ohashi, 2002; Viola et al., 2011; Manassero et al., 2013). The different binding preferences between the two classes are dependent on the presence of glycine or aspartic acid at positions 11 or 15, respectively (Viola et al., 2011; Danisman, 2016). TCP transcription factors can form homodimer or heterodimers to bind DNA sequences of target genes, and their dimerization was first described by the interaction of PCF1 and PCF2 in rice (Kosugi and Ohashi, 1997). Later, protein-protein interactions between *Arabidopsis* TCP transcription factors were provided to demonstrate that TCP proteins from the same class showed a preference to interact with each other (Danisman et al., 2013). Moreover, TCP proteins can also interact with a variety of other proteins, such as immune adaptor SRFR1, components of the circadian clock (TOC1, CCA1, LHY, PRR3, and PRR5), transcriptional repressor TIE1, histidine-containing phosphotransmitters (AHP1, AHP2, and AHP3), and phytoplasma protein effector SAP11 (Pruneda-Paz et al., 2009; Giraud et al., 2010; Sugio et al., 2011; Tao et al., 2013).

*TCP* family genes have been identified with different numbers in different plants, such as 24 members in *Arabidopsis thaliana* (Danisman et al., 2013), 22 in *Oryza sativa* (Cubas et al., 1999; Xu et al., 2017), 30 in *Solanum lycopersicum* (Parapunova et al., 2014), 17 in *Camellia sinensis* (Wu et al., 2017), and 33 in *Populus euphratica* (Ma et al., 2016). Generally, TCP transcription factors have a conserved role as regulators for branching, floral asymmetry, and cell proliferation (Luo et al., 1996; Doebley et al., 1997; Kosugi and Ohashi, 1997). A classic example is *TB1* and its homologs that regulate the architectures of a branch, shoot, and/or inflorescence in maize, rice, wheat, and *Arabidopsis* (Doebley et al., 1997; Lewis et al., 2008; Dixon et al., 2018). Besides, multiple studies showed that *TCP* genes are also involved in leaf development, gametophyte development, seed germination, regulation of circadian clock, and influencing of hormone pathways (Takeda et al., 2006; Tatematsu et al., 2008; Pruneda-Paz et al., 2009; Sarvepalli and Nath, 2011; Rueda-Romero et al., 2012; Aguilar-Martínez and Sinha, 2013; Manassero et al., 2013). Moreover, some studies suggested that the TCP-controlled growth responses could also be linked with environmental challenges, such as plant pathogen, high light, salt, and nutritional stresses (Mukhtar et al., 2011; Guan et al., 2014; Mukhopadhyay and Tyagi, 2015; Danisman, 2016; Viola et al., 2016). However, evidence for the innovation and the emerging role of *TCP* family genes in abiotic stress has been lacking.

With the completion of genome sequencing, *Populus trichocarpa* has been considered to be an ideal model species for genomic and genetic studies of woody plants (Tuskan et al., 2006). In this study, we visited *PtrTCP* family genes and traced their evolutionary trajectory, especially their expansion experience around K-Pg extinction event followed by the Cenozoic global cooling. Subsequently, we investigated the family expression profile in different tissues under different abiotic stresses and identified a cold-associated regulatory module including *PtrTCP31*, *PtrTCP10*, and *PtrTCP36*. Accordingly, we selected a rWGD-duplicated and cold-induced *PtrTCP* gene (*PtrTCP10*) to study its potential role under abiotic stresses. Our results provide new insights into the studies of *TCP* genes in woody plants, and they may serve as an impetus to explore the molecular mechanism of *TCP* genes in response to environmental stresses.

## Materials and Methods

### Identification of TEOSINTE BRANCHED1/CYCLOIDEA/PROLIFERATING CELL FACTOR Transcription Factors in *P. trichocarpa*

The genome sequences of *Arabidopsis* and *P. trichocarpa* were downloaded from TAIR10 (Huala et al., 2001) and Phytozome v13.1 (Goodstein et al., 2012), respectively. To obtain TCP homologs in *P. trichocarpa*, we first used the 24 *Arabidopsis* TCP proteins that have been genome-wide identified (Martín-Trillo and Cubas, 2010) as query sequences to perform BLASTP against *P. trichocarpa* proteome sequences (*P. trichocarpa* v3.1), and those genes with *E*-value < 1e-10 were selected as candidate PtrTCP proteins. In addition, we also downloaded the predicted *P. trichocarpa* TCP proteins from the Plant Transcription Factor Database (PlantTFDB 5.0) (Tian et al., 2020). Based on the above sequences, we further confirmed TCP domain containing proteins using hmmsearch^1^ using the Hidden Markov Model (HMM) profile of TCP domain (PF03634) (Finn et al., 2014, 2016). Accordingly, we obtained a total of 36 *PtrTCP* genes in *P. trichocarpa*.

### Phylogenetic Analysis, Gene Structure, and Conserved Motif Analysis

The TCP proteins of *P. trichocarpa* and *Arabidopsis* were aligned by MUSCLE program (Edgar, 2004). The phylogenetic tree was constructed using the maximum-likelihood (ML) method with the bootstrap of 1,000 implemented in IQ-TREE (Nguyen et al., 2015). Exon/intron information of *TCP* family genes was obtained from gene annotation files of *P. trichocarpa* and *Arabidopsis*. To investigate the conserved functional domain of PtrTCP proteins, we downloaded the HMMs of all protein domains from the Pfam database (Finn et al., 2016) and used hmmsearch (see text footnote 1) to search TCP proteins against the HMMs with an *E*-value < 1e-5. The locations of the conserved functional domains across the TCP proteins were further visualized using TBtools version 1.0971 (Chen et al., 2020).

### Synteny Analysis and Expansion History of *PtrTCP* Genes

To trace the expansion history of *PtrTCP* genes, MCScanX (Wang et al., 2012) was utilized to identify collinear gene blocks, some of which contained *PtrTCP* genes, and the synteny analysis result was visualized using the Circos software in TBtools version 1.0971 (Chen et al., 2020). In dating the expansion of *PtrTCP* family genes, the synonymous substitution (*Ks*) value of each pair of the collinear gene blocks was calculated using the YN method by KaKs_Calculator2.0 (Wang et al., 2010). Similarly, the average *Ks* of all the collinear gene blocks between *P. trichocarpa* and *Arabidopsis* was also calculated.

### Gene Ontology Enrichment and *Cis*-Regulatory Element Analysis

Gene ontology (GO) term assignment for *P. trichocarpa* genes was obtained using the eggnog-mapper version 2 tool, and GO term enrichment analysis of *PtrTCP* genes was performed using GOSeq (Young et al., 2010; Cantalapiedra et al., 2021). The GO terms with a value of *p* < 0.05 were considered to be significantly enriched. To identify potential *cis*-regulatory elements within the promoter sequences of *PtrTCP* genes, we searched 1-kb sequence upstream of the translation initiation site (TIS) of *PtrTCP* genes in PlantCARE (Rombauts et al., 1999; Lescot et al., 2002) and visualized the distribution of the enriched elements using TBtools version 1.0971 (Chen et al., 2020).

### RNA-Seq Analysis of *P. trichocarpa* Leaf, Stem, and Root Samples Under Abiotic Stresses

Transcriptome data of *P. trichocarpa* under abiotic stresses were downloaded from the NCBI BioProject (accession ID: PRJEB19784) (Filichkin et al., 2018). Trimmomatic software was utilized to remove the Illumina adapter contamination and filter the low-quality bases (Bolger et al., 2014), and then, the obtained clean reads were mapped to *P. trichocarpa* genome (version 3.1) by HISAT2 (Pertea et al., 2016). StringTie was further utilized to calculate the Transcripts Per Kilobase Million (TPM) value for each gene in the genome (Pertea et al., 2016). According to the gene expression, we calculated Pearson correlation coefficients of the leaf, stem, and root samples under cold, heat, salt, and drought stresses using R programming and visualized the expression patterns of *PtrTCP* genes using TBtools v1.0971 (Chen et al., 2020). The differentially expressed genes (DEGs) between two groups were obtained using edgeR, DESeq2, and Ballgown (Robinson et al., 2010; Trapnell et al., 2013; Love et al., 2014; Frazee et al., 2015). Those genes that had an adjusted *p* < 0.05 and an absolute value of fold change ≥2 from at least two of the methods were considered to be DEGs. Finally, we obtained the DEGs of *PtrTCP* genes in *P. trichocarpa* leaf, stem, and root samples under cold, heat, salt, and drought stresses.

### Validation of *PtrTCP* Genes in Response to Abiotic Stresses by Quantitative Real-Time PCR

*Populus trichocarpa* seedlings were cultured in an artificial climate chamber with 25°C at a photoperiod of 16/8 h light/dark cycle, and the 2-month-old *P. trichocarpa* seedlings with a similar growth status were utilized for abiotic stress treatments, including cold (4°C), heat (39°C), salt (200 mM NaCl), and drought (20% PEG6000). The fourth expanded leaves of *P. trichocarpa* seedlings were separately collected after short period (24 h for cold, salt, and drought, while 12 h for heat) and long period (7 days) of the corresponding abiotic stress treatments. Total RNA was extracted using the TRIzol reagent (Invitrogen, Carlsbad, CA, United States) following the manufacturer’s procedure, and cDNA was synthesized by the PrimeScript RT™ Reagent Kit with gDNA Eraser (TaKaRa, Japan). Quantitative Real-time PCR (qRT-PCR) was performed using a CFX96 real-time PCR system with ChamQ SYBR qPCR Master Mix (Vazyme, Nanjing, China), and PCR reaction was performed under the following conditions: 95°C for 10 min, 45 cycles of 95°C for 30 s, and 60°C for 10 s. Relative expression level of each gene was normalized to *PtrHIS* (*Potri.005G072300*), *Ptr60S* (*Potri.007G093700*), *PtrACTIN* (*Potri.019G010400*), and *PtrACTIN2* (*Potri.001G309500*), and the achieved data were analyzed using the 2^–ΔΔCT^ method (Livak and Schmittgen, 2001).

### Gene Co-expression Network Analysis

Based on the above RNA-seq analysis of *P. trichocarpa* under abiotic stresses, R package WGCNA was utilized to identify modules of highly correlated genes that were associated with each abiotic stress treatment (Langfelder and Horvath, 2008). First, Pearson’s correlation coefficients were calculated for all pair-wise genes, and soft threshold was then obtained to construct an adjacency matrix. Subsequently, the topological overlap matrix (TOM) was calculated from the resulting adjacency matrix, and the genes were hierarchically clustered based on TOMsimilarity. Finally, the dynamic tree cut algorithm was utilized to cut the hierarchal clustering tree into gene modules (minModuleSize = 10 and cutHeight = 0.2), and the Pearson’s correlation coefficients between modules and abiotic stresses were calculated.

### Generation of 35S::*PtrTCP10* Transgenic Lines in *Arabidopsis*

The full length of *PtrTCP10* coding sequence (CDS) without the stop codon was amplified from the wild-type *P. trichocarpa* cDNA using the *PtrTCP10* specific primers designed with *Kpn*I and *Sal*I sites, and then, the purified PCR product was inserted into the pCAMBIA1300-sGFP vector under the control of CaMV35S promoter. After transferring pCAMBIA1300-*PtrTCP10*-sGFP plasmid into *Agrobacterium tumefaciens* EHA105 competent cells, transformation of *PtrTCP10* into *Arabidopsis* was further performed using the floral dip method. Finally, the positive seedlings screened on the hygromycin-resistant plates were selected as candidate 35S::*PtrTCP10* overexpression (OE) lines, and each of the candidate 35S::*PtrTCP10* OE lines was verified by PCR and qRT-PCR.

### Freezing Tolerance Assay

For freezing tolerance assay, 3-week-old plotted cultivation 35S::*PtrTCP10* OE lines and wild-type plant (Col-0) that were cultured under a 12/12 h light/dark photoperiod were treated with or without cold acclimation (4°C for 3 days), and then, freezing tolerance assays were performed as described by Jia et al. (2016). In brief, the program was set at 4°C for 10 min and 0°C for 20 min and dropped 1°C/h to the desired temperatures. After freezing treatment, the plants were grown at 4°C in dark for 12 h and then were transferred to 22°C for additional 3 days. Freezing tolerance phenotype of each line was observed, and the survival rate of each line was also calculated.

### Salt Stress Assay

For salt stress assay, seeds of the 35S::*PtrTCP10* OE lines and wild-type plant (Col-0) were cultured on the 1/2 MS media containing different concentrations of NaCl (0, 100, 125, and 150 mM). After being treated at 4°C for 2 days, seeds were cultured in a light incubator with 23°C at a photoperiod of 12/12 h light/dark cycle. The number of the germinated seeds of each line under different salt stress treatments was counted every day, and the germination rate of each line was finally calculated. In addition, we also measured root length and fresh weight of the salt-treated seedlings.

### Construction of PtrTCP10-Mediated Regulatory Network

The Pearson correlation coefficient (PCC) between *PtrTCP10* and other genes was calculated by using the above transcriptome data of *P. trichocarpa* under cold and salt stresses, and the genes with |PCC| > 0.7 and *p* < 0.0001 were identified as *PtrTCP10* co-expressed genes. GO enrichment analysis of the co-expressed genes was performed and visualized by ClueGO (Bindea et al., 2009). To further predict the putative target genes of PtrTCP10, 1-kb sequence upstream of the TIS of *PtrTCP10* co-expressed genes was utilized to search for PtrTCP10 binding site with at most one-mismatch across the consensus sequence GTGGNCCC. Accordingly, a regulatory network centrally mediated by PtrTCP10 was constructed based on PtrTCP10 and its putative co-expressed target genes that contained at least two TCP binding sites. In addition, the well-known cold- and salt-relevant genes (e.g., *ZAT10*, *GolS2*, *HY5*, *CBL1*, and *SOS1*) that contained one TCP binding site were also included in this regulatory network (Wu et al., 1996; Cheong et al., 2003; Sakamoto et al., 2004; Nishizawa et al., 2008; Zhang et al., 2020).

## Results

### Exon/Intron Structures and Protein Domains of *PtrTCP* Family Show Significant Conservation but Also Evolutionary Divergence

**FIGURE 1:**
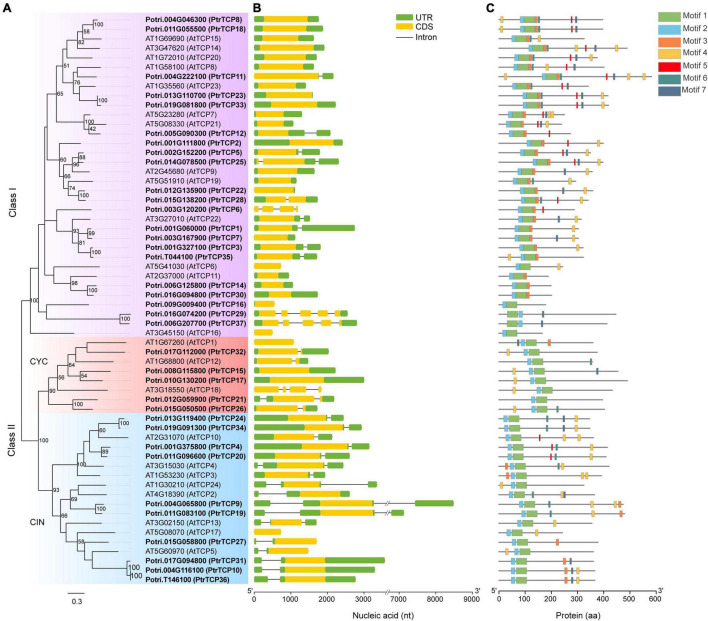
Phylogenetic analysis and architecture of TEOSINTE BRANCHED1/CYCLOIDEA/PROLIFERATING CELL FACTOR (*TCP*) family genes in *Populus trichocarpa* and *Arabidopsis*. **(A)** Phylogenetic analysis of *TCP* family genes. The amino acid sequences of *P. trichocarpa* (in bold) and *Arabidopsis* TCP proteins were aligned to construct the phylogenetic tree using maximum-likelihood (ML) method with 1,000 bootstrap replicates. Genes clustered in the same subfamily are marked by the same color. **(B)** Exon/intron structures of *TCP* family genes. Exon and intron are represented by a colored rectangle and black line, respectively. Of the exon, the yellow rectangle represents coding sequence (CDS) and the green rectangle represents 5′ UTR or 3′ UTR. **(C)** Distribution of the conserved domains or motifs in TCP proteins. Different kinds of conserved motifs are indicated by the rectangles with different colors.

Based on a homolog search in combination with previous reported *PtrTCP* genes (Ma et al., 2016), we gave an updated list of the family, designating *PtrTCP1* to *PtrTCP12* and *PtrTCP14* to *PtrTCP37*. As a total, we obtained 36 *PtrTCP* genes (Supplementary Table 1), which showed 50% more than that in *Arabidopsis* (Danisman et al., 2013). Among them, *PtrTCP37* (*Potri.006G207700*) was a newly identified member that had not been reported previously, and a 41-amino acid length protein identified as *PtrTCP13* (*Potri.005G140600*) by Ma et al. (2016) was excluded. To elucidate phylogenetic relations of *PtrTCP* family, we constructed a phylogenetic tree by including *Arabidopsis TCP* genes as references (Figure 1A). The tree showed that *PtrTCP* genes can be classified into two major groups (i.e., class I and class II), and the members within class II were further divided into two lineages (i.e., CIN and CYC), supporting the previous results from other plants (Kosugi and Ohashi, 2002; Navaud et al., 2007; Li, 2015).

As the gene structure is considered to provide important clues for the conservation and evolution of homologous genes (Xu et al., 2017), we analyzed the gene structures of *PtrTCP* genes (Figure 1B). *PtrTCP* genes were likely to have relatively simple exon/intron structures, with the exon number from one to five, and most of the *PtrTCP* genes within the same subfamily shared similar gene structures. Approximately, 86% of *PtrTCP* genes contained one or two exons, while only two genes (i.e., *PtrTCP29* and *PtrTCP37*) contained five exons. In addition, we also detected the conserved domains or motifs of PtrTCP proteins, and a total of seven conserved motifs were identified (Figure 1C and Supplementary Figure 1). Of the motifs, motifs 1 and 2 constituted the TCP domain. Interestingly, PtrTCP29 and PtrTCP37 have an order of motif 1 followed by motif 2 in contrast to all of the other PtrTCP proteins sharing an order of motifs 2 and 1, demonstrating that PtrTCP29 and PtrTCP37 underwent an evolution of reverse positions of motifs 1 and 2. Except for the two motifs, it is worthy to note that motif 4 with an enrichment of Gln (Q) is the most widely distributed in the PtrTCP proteins (Figure 1C and Supplementary Figure 1). Together, *PtrTCP* genes have a similar composition of exon/intron structures and protein domains but also some divergences especially between the subfamilies.

### Three Whole Genome Duplication Events Are Responsible for the Innovation of *PtTCP* Family Genes

**FIGURE 2:**
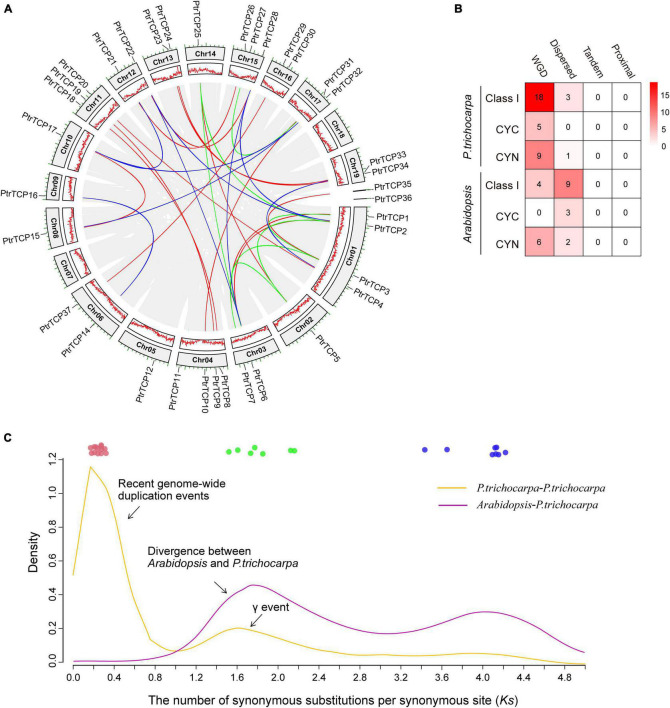
Chromosomal distribution and expansion mechanisms of *PtrTCP* family genes. **(A)** Chromosomal distribution and collinear blocks of *PtrTCP* family genes. The collinear gene pairs of *PtrTCP* genes are marked with different colors (blue, green, and red) according to the three independent whole genome duplication (WGD) events (ancient WGD, γ-WGT, and rWGD). **(B)** Comparison of the duplication mechanisms in producing *TCP* family genes between *P. trichocarpa and Arabidopsis*. **(C)** Density distribution of the *Ks* between all collinear gene blocks within *P. trichocarpa* and between *P. trichocarpa and Arabidopsis*. The colored circles indicate the collinear gene blocks containing *PtrTCP* genes, and they are marked with different colors (blue, green, and red) based on the three main WGD events (ancient WGD, γ-WGT, and rWGD*).*

As described above, *P. trichocarpa* has 50% more *TCP* genes than *Arabidopsis.* To investigate the innovation of *PtrTCP* family genes, we detected their chromosomal distribution and gene duplication events. *PtrTCP* genes were distributed unequally on *P. trichocarpa* chromosomes, with 34 genes on 17 chromosomes and two genes on the unmapped scaffolds (Figure 2A). Intra-specie genomic comparison generated hundreds of collinear gene blocks; Of which, 30 blocks were identified to contain 32 *PtrTCP* genes (Figure 2A, gene block pairs connected by color lines). Accordingly, WGD is the major duplication mechanism to generate *PtrTCP* genes in *P. trichocarpa* (Figure 2B).

To further estimate the time of WGD-expanded *PtrTCP* genes, we calculated the synonymous substitutions per synonymous site (*Ks*) of collinear gene blocks (Supplementary Table 2). We found that the gene blocks could be mainly classified into three categories (Figure 2C). The first category included nine pairs of *PtrTCP* genes (shown in blue) with *Ks* value ranging from 3.43 to 4.66, which were derived from the ancient angiosperm-wide or seed plant-wide WGD (Jiao et al., 2011). The second category included seven pairs of *PtrTCP* genes (shown in green) that were derived from the γ whole-genome triplication (γ-WGT) shared by core eudicots (Tang et al., 2008). The third category included 14 pairs of *PtrTCP* genes (shown in red) with *Ks* value ranging from 0.17 to 0.31, suggesting that these genes blocks were originated from the most recent WGD (rWGD) in *Populus* lineage after its divergence from *Arabidopsis*, which explained well 50% more *PtrTCP* genes in *P. trichocarpa* than that in *Arabidopsis*. The findings demonstrated that the three WGD events are the dominant underlying mechanism for expanding *PtrTCP* family genes, and rWGD is the main event to lead the innovation of *PtrTCP* family genes in contrast to *Arabidopsis*.

Interestingly, the rWGD event occurred around K-Pg extinction event ∼66 million years ago, which was followed by the Cenozoic global cooling (Wu et al., 2020). Those rWGD-duplicated *PtTCP* genes might have undergone subfunctionalization or neofunctionalization, which contributed to their retention and plant adaptation during the cooling stress. Then, we investigated the functional enrichments of *PtTCP* genes and their regulation under abiotic stresses, especially cold stress.

### Enrichments of Gene Ontology Terms and *Cis*-Regulatory Elements Reveal That *PtrTCP* Genes Might Be Involved in Abiotic Stresses

**FIGURE 3:**
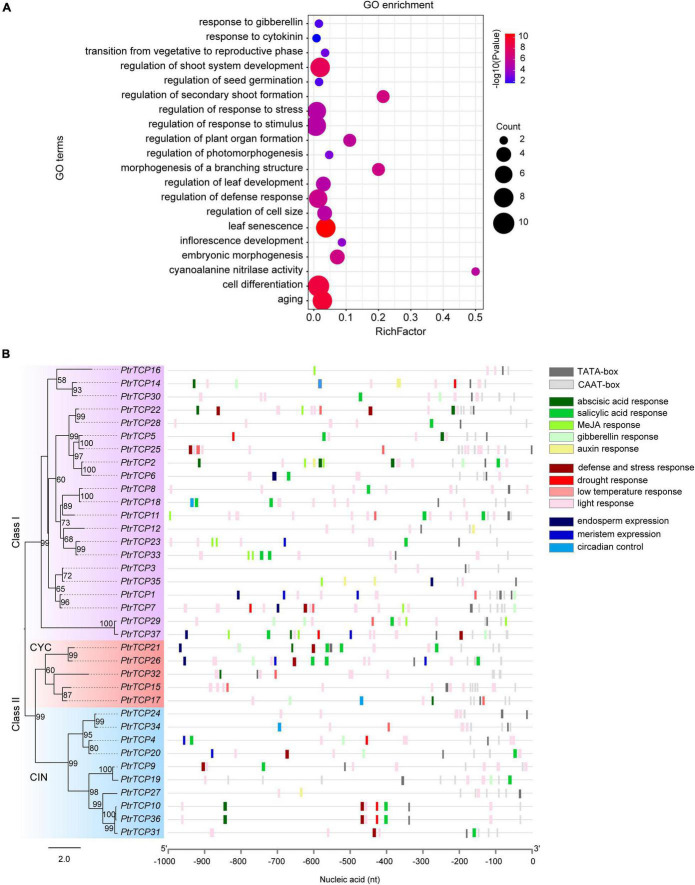
GO enrichment and *cis*-regulatory element analysis of *PtrTCP* family genes. **(A)** Gene ontology (GO) enrichment analysis of *PtrTCP* family genes. The dot size represents the number of enriched genes, and the colored bar represents the significant enrichment level of GO term. **(B)** Distribution of the *cis*-regulatory elements in the 1-kb promoter sequence upstream of the translation initiation site (TIS) of *PtrTCP* genes.

To investigate the biological pathways that *PtrTCP* genes might participate in, GO term enrichment analysis of *PtrTCP* genes was performed (Figure 3A). We found that the GO terms related to the regulation of various developmental processes were significantly enriched, such as shoot system development, morphogenesis of a branching structure, timing of transition from vegetative to reproductive phase, inflorescence development, seed germination, and embryonic morphogenesis, which were consistent with previous studies (Takeda et al., 2006; Tatematsu et al., 2008; Sarvepalli and Nath, 2011; Rueda-Romero et al., 2012; Aguilar-Martínez and Sinha, 2013; Manassero et al., 2013). In addition, GO terms related to the regulation of response to hormone and environmental stresses were also enriched, such as response to gibberellin, response to cytokinin, regulation of response to stress, and regulation of response to stimulus. These results indicated that *PtrTCP* genes may not only be involved in plant growth and development but also participate in response to hormones and environmental stresses.

*Cis*-regulatory elements in promoters are essential for regulating gene expression (Lescot et al., 2002). We predicted potential *cis*-regulatory elements in the 1-kb sequence upstream of TIS of *PtrTCP* genes using PlantCARE (Rombauts et al., 1999). In addition to the common *cis*-regulatory elements (e.g., TATA-box and CAAT-box), we found *cis*-regulatory elements in relation to plant growth and development (e.g., endosperm expression, meristem expression, and circadian control), and in response to environmental stresses (e.g., low-temperature, drought, and light) and hormone responses (e.g., abscisic acid, salicylic acid, MeJA, gibberellin, and auxin) (Figure 3B). This suggested that the expression of *PtrTCP* genes might be dynamically regulated in plant growth and development under normal and/or environmental stresses. Then, we investigated the expression profiles of *PtrTCP* genes in leaf, stem, and root tissues under normal and stress conditions (e.g., cold, heat, salt, and drought).

### Expression Profiles of *PtrTCP* Subfamilies Are Diversified in Leaf, Stem, and Root Tissues

**FIGURE 4:**
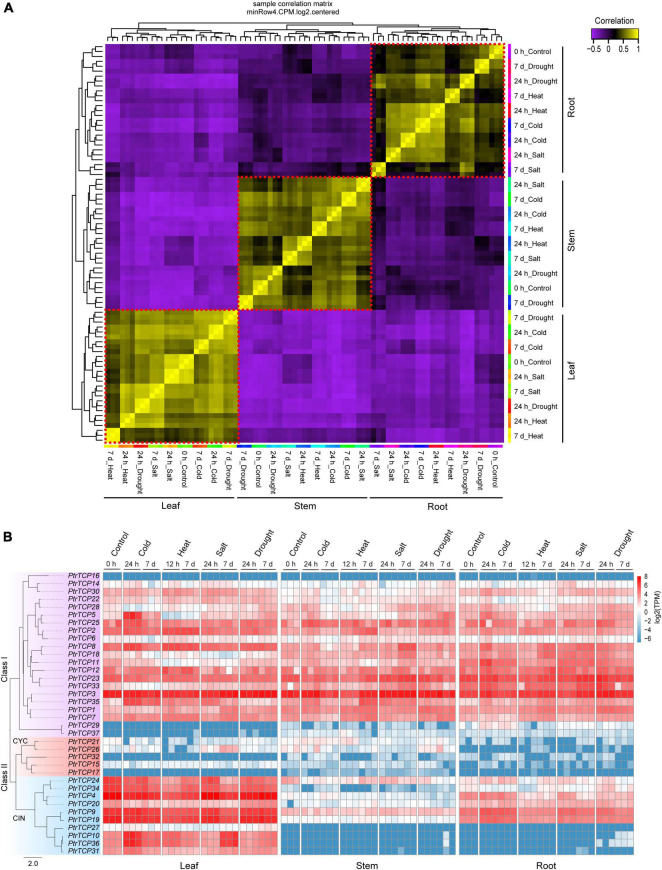
Transcriptional expression patterns of PtrTCP genes in different tissues under cold, heat, salt, and drought stresses. **(A)** Correlation heat map of the samples based on transcriptome-scale expressed genes. Three clear clusters in red dotted boxes were obtained in consistence with root, stem, and leaf samples. **(B)** Expression heat map of *PtrTCP* genes. The values of transcripts per million reads (TPM) were used to represent the expression levels of *PtrTCP* genes.

Correlation analysis on transcriptome-scale gene expression from *P. trichocarpa* samples showed three clusters, which were classified by leaf, stem, and root tissues rather than by abiotic stresses (Figure 4A). This demonstrated that the tissue type was the main factor to classify the samples, and meanwhile, the genes in these tissues were likely to be elaborately controlled in response to abiotic stresses. Inspecting the expression profiles of *PtrTCP* genes in the tissues under abiotic stresses (Figure 4B), we found that most of the class I genes were highly expressed, while the genes in CYC lineage were lowly expressed in the three tissues despite all of the abiotic stresses. However, *PtrTCP* genes in CIN lineage showed diversified expression profiles. In detail, 50% of CIN lineage genes including *PtrTCP24*, *PtrTCP34*, *PtrTCP4*, *PtrTCP9*, and *PtrTCP19* were highly, moderately, and lowly expressed, respectively, in leaf, root, and stem, and some CIN lineage genes (e.g., *PtrTCP10*, *PtrTCP36*, *PtrTCP31*, and *PtrTCP27*) were especially expressed in leaf tissue, suggesting an important role of CIN lineage in leaves. Moreover, *PtrTCP* genes were likely to be elaborately regulated in response to abiotic stresses (Figure 4B). For example, *PtrTCP10*, *PtrTCP36*, *PtrTCP35*, and *PtrTCP5* were obviously upregulated in leaves after cold stress. Then, we investigated the abiotic stress-affected *PtrTCP* genes in detail.

### The Emerging Roles of *PtrTCP* Genes in Response to Abiotic Stresses

**FIGURE 5:**
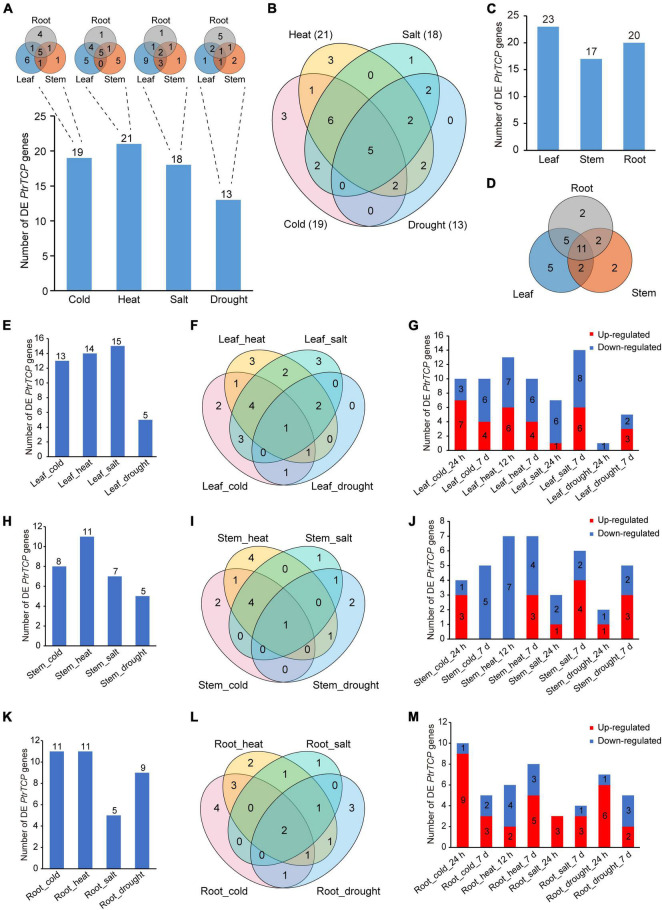
Identification of the differentially expressed (DE) *PtrTCP* genes that could significantly respond to abiotic stresses. **(A,B)** The DE *PtrTCP* genes in response to different abiotic stresses. **(C,D)** The DE *PtrTCP* genes in response to abiotic stresses in different tissues. **(E–M)** The DE *PtrTCP* genes in response to abiotic stresses in leaf **(E–G)**, stem **(H–J)**, and root **(K–M)**.

We compared the expression changes of *PtrTCP* genes in the tissues between abiotic stresses and normal condition (Figure 5 and Supplementary Table 3). In the aspect of *PtrTCP* genes under each of the four abiotic stresses, there were 19, 21, 18, and 13 differentially expressed (DE) ones, respectively, in response to cold, heat, salt, and drought stresses. Under the same stress, different tissues showed an overlap but also a great divergence of DE *PtrTCP* genes (Figure 5A). For cold, heat, and salt stresses, most of the DE *PtrTCP* genes were determined in leaf, while for drought, the counterpart was determined in the root. In addition, some of those *PtrTCP* genes could be induced by multiple abiotic stresses. For example, 22 genes could respond to at least two of the stresses, and in particular, five genes were differentially regulated by all the four abiotic stresses (Figure 5B). In the aspect of *PtrTCP* genes in each of the tissues, 23, 17, and 20 genes were differentially regulated by at least one of the four abiotic stresses, respectively, in leaf, root, and stem (Figure 5C). Of them, 11 DE *PtrTCP* genes could respond to abiotic stresses in the three tissues (Figure 5D). In leaf, 13, 14, 15, and 5 *PtrTCP* genes were DE under the treatment of cold, heat, drought, and salt, respectively (Figure 5E). In addition to the overlaps of DE *PtrTCP* genes between the abiotic stresses, some *PtrTCP* genes could uniquely respond to one of cold, heat, and salt in leaf (Figure 5F). Moreover, of the DE *PtrTCP* genes, some showed different expression patterns in response to short periods and/or long periods of abiotic stresses (Figure 5G and Supplementary Table 3). Likewise, in stem and root, we also observed similar patterns of DE *PtrTCP* genes in response to abiotic stresses of cold, heat, salt, and drought at different time points (Figures 5H–M).

**FIGURE 6:**
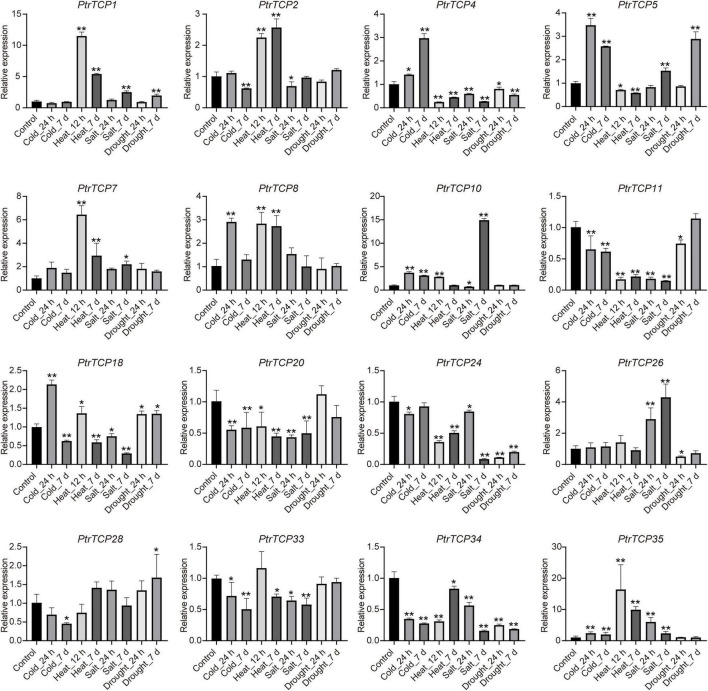
qRT-PCR validation of the selected *PtrTCP* genes in leaf response to abiotic stresses. The relative expression level of each *PtrTCP* gene was normalized to *PtrHIS (Potri.005G072300), Ptr60S (Potri.007G093700), PtrACTIN (Potri.019G010400)*, and *PtrACTIN2 (Potri.001G309500)*. The expression values are the means of three technical replicates, and asterisks represent a significant difference between each stress treatment and negative control (*p < 0.05, **p < 0.01; Student’s *t*-test). Each qRT-PCR experiment was performed with three independent biological replicates, and the error bars represent SD.

To further validate the expression of *PtrTCP* genes in response to abiotic stresses, 16 DE *PtrTCP* genes that could be induced or repressed by at least one of the abiotic stresses were selected to be for experimentally qRT-PCR confirmation in leaf (Supplementary Table 4). Except for *PtrTCP33*, the selected *PtrTCP* genes could respond to at least one of the abiotic stresses, showing similar expression trends under abiotic stresses with those detected by RNA-seq (Figure 6 and Supplementary Table 3). For example, *PtrTCP5*, *PtrTCP10*, and *PtrTCP35* were significantly upregulated by cold treatments at 24 h and 7 days, while *PtrTCP34* was downregulated. *PtrTCP1*, *PtrTCP4*, *PtrTCP2*, *PtrTCP7*, *PtrTCP8*, *PtrTCP11*, and *PtrTCP35* responded to both the short period and the long period of heat treatments. *PtrTCP1*, *PtrTCP7*, *PtrTCP10*, *PtrTCP26*, and *PtrTCP35* were significantly induced by salt stress, and in contrast, *PtrTCP4*, *PtrTCP11*, *PtrTCP18*, *PtrTCP20*, *PtrTCP24*, and *PtrTCP34* were repressed. These findings suggested that at least half of the *PtrTCP* genes could respond to abiotic stresses, and they might be involved in regulating abiotic stress tolerance. Then, we sought to explore the potential hub *PtrTCP* genes in an abiotic process using the gene co-expression network.

### Co-expression Network Identifies a Cold-Associated Module Including Three *PtrTCP* Genes (*PtrTCP10*, *PtrTCP31*, and *PtrTCP36*)

**FIGURE 7:**
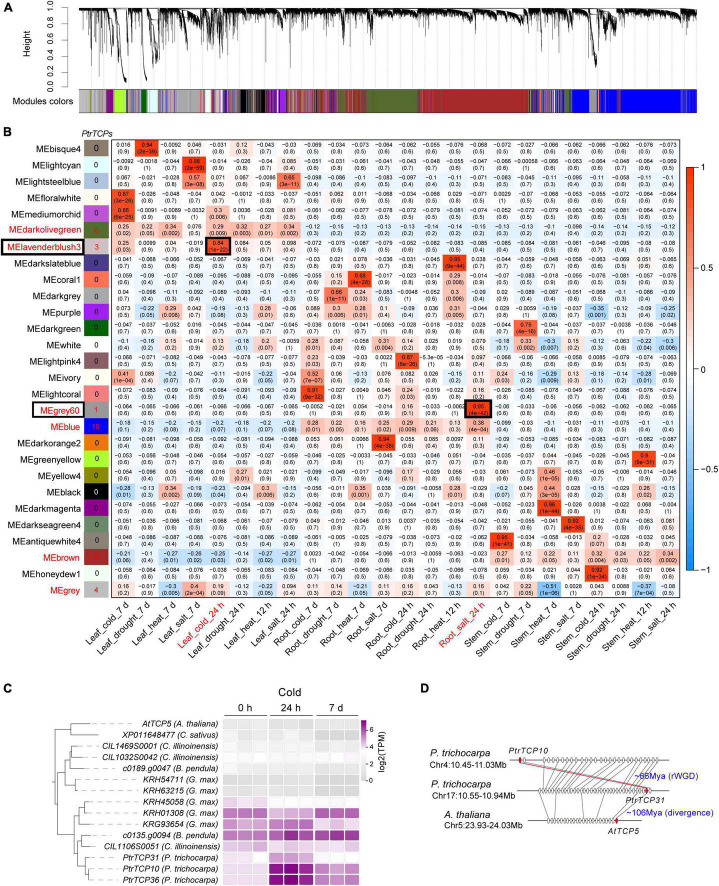
Distribution of *PtrTCP* genes in the regulatory modules of *P. trichocarpa* tissues under abiotic stresses. **(A)** Gene regulatory modules identified by weighted gene co-expression network analysis (WGCNA). **(B)** Relationships of module eigengenes (ME, rows) and traits of the tissues under abiotic stresses (columns). Red/blue represents positive/negative correlations between the modules and the traits. The six modules containing *PtrTCP* genes were marked in red, and the number of *PtrTCP* genes was shown nearby. Of the six modules, the ones significantly correlated with one of the traits were especially shown in the box, and the corresponding traits were colored in red. **(C)** Expression conservation and divergence *of PtrTCP31/10/36* orthologs in dicots under cold stress at different time points (0 h, 24 h, and 7 days). The cold-treated samples were from *Arabidopsis thaliana (A. thaliana), Cucumis sativus (C. sativus), Carya illinoinensis (C. illinoinensis), Betula pendula (B. pendula), Glycine max (G. max)*, and *Populus trichocarpa (P. trichocarpa)*. **(D)** The generation process of *PtrTCP10* gene. *PtrTCP10* was rWGD-duplicated from *PtrTCP31* and underwent an inversion, where the *P. trichocarpa* region containing *PtrTCP31* (Chr17:10.55–10.94) showed collinear orthologous gene pairs with *Arabidopsis* homologous region (Chr5:23.93–24.03 Mb) containing *AtTCP5.*

Based on the expressed genes including *PtrT* in leaf, root, and stem under abiotic stresses, we constructed a co-expression network (Figure 7A) using weighted correlation network analysis (WGCNA; Langfelder and Horvath, 2008). In the network, 28 modules were obtained, and some modules were significantly correlated with the tissue traits (Figure 7B). For example, the module MElavenderblush3 was significantly positively correlated with the trait (Leaf_cold_24h), which indicated that the genes in MElavenderblush3 were especially upregulated in the same orientation in leaf under cold stress at 24 h. In the case of *PtrTCP* family genes, 30 members were clustered into six modules, e.g., ten in MEblue, six in MEbrown, six in MEdarkolivegreen, four in MEgrey, three in MElavenderblush3, and one in MEgrey60 (Figure 7B and Supplementary Table 5). However, of the six modules, only two modules (i.e., MEgrey60 and MElavenderblush3) including four *PtrTCP* genes were significantly correlated with abiotic stress treatments. Although *PtrTCP12* in MEgrey60 was correlated with Root_salt_24h, the gene was not significantly upregulated (<2 fold change) by salt treatment at 24 h (Supplementary Table 3). In contrast, *PtrTCP10*, *PtrTCP31*, and *PtrTCP36* in MElavenderblush3 were correlated and significantly upregulated in leaf under cold stress at 24 h (Leaf_cold_24h), demonstrating a potential role of the genes in MElavenderblush3 under cold stress.

Notably, *PtrTCP10*, *PtrTCP31*, and *PtrTCP36* genes were *P. trichocarpa* inparalogs related to other dicot orthologs including *Arabidopsis* singular ortholog *AtTCP5* and *Glycine* five co-orthologs (the left panel in Figure 7C). This demonstrated the three genes evolved from gene duplications within *P. trichocarpa* lineage after the divergence from other dicot lineages, which was consistent with the above WGD analysis (Figure 2). Further expression analysis of those genes showed that *PtrTCP10* and *PtrTCP36* were significantly upregulated (>5-fold change) by cold stress, whereas the corresponding co-orthologs in other close dicots (e.g., *Arabidopsis*, *Cucumis sativus*, *Carya illinoinensis*, *Betula pendula*, and *Glycine max*) were not or less affected (the right panel in Figure 7C and Supplementary Table 6). Based on the expansion history of *PtrTCP* genes (Figure 2), *PtrTCP36* was mapped on a scaffold and predicated to be produced by dispersed duplication from *PtrTCP10*, whereas *PtrTCP10* was mapped on Chr4 and duplicated from *PtrTCP31* by rWGD ∼66 million years ago (Figure 7D). After rWGD, the Cenozoic global cooling and other environmental factors might have driven subfunctionalization and/or neofunctionalization of these *TCP* genes in regulation and function, and of those *TCP* orthologs in the six plants, only *PtrTCP10* and *PtrTCP36* acquired a strong capability of cold induction. We questioned whether the acquirement of cold-induced capability in *PtrTCP10* and *PtrTCP36* contributed to the cold adaptation of the plants. Then, we selected the WGD-duplicated *PtrTCP10* for OE in *Arabidopsis* to investigate its potential role in cold stress.

### Overexpression of *PtrTCP10* Increases Freezing Tolerance and Salt Sensitivity

**FIGURE 8:**
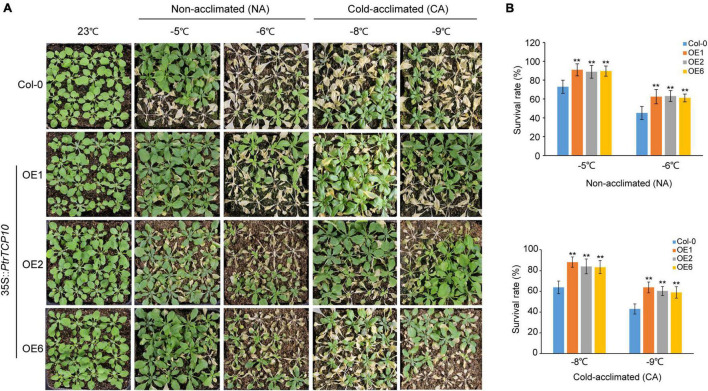
Overexpression of *PtrTCP10* in *Arabidopsis* increased freezing tolerance. **(A)** Comparison of the freezing tolerance phenotypes between 35S*::PtrTCP10* OE lines and wild-type (Col-0) under freezing treatments with or without cold acclimation. **(B)** Survival rate of the 35S::*PtrTCP10* OE lines and wild type after freezing treatments for 3 days. The values are the means of at least three replicates, and the error bars represent SD. Asterisks represent a significant difference between 35S::*PtrTCP10* OE lines and wild-type (**p < 0.01; Student’s *t*-test).

**FIGURE 9:**
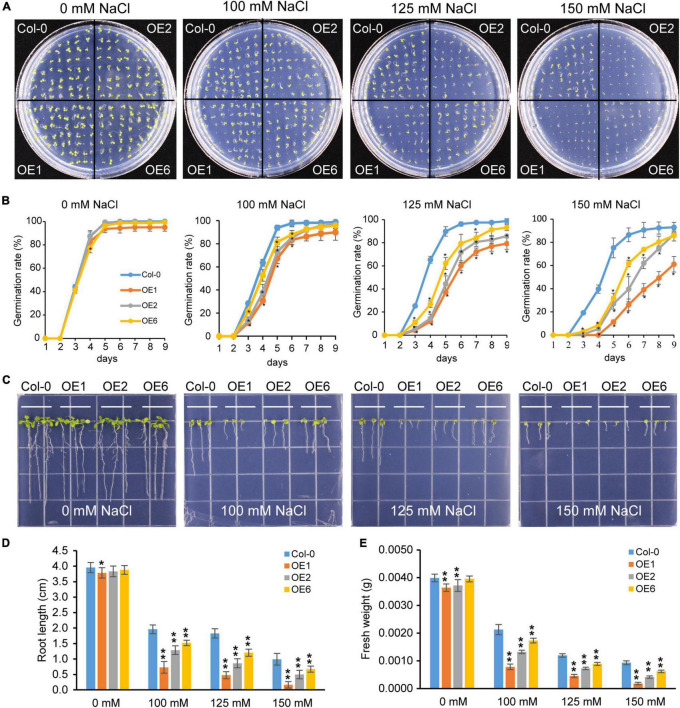
Overexpression of *PtrTCP10* increased salt sensitiveness. **(A)** Comparison of the seed germination phenotypes between 35S::*PtrTCP10* OE lines and wild-type (Col-0) under salt stresses with different concentrations of NaCl. **(B)** Germination rate of 35S::*PtrTCP10* OE lines and wild type under salt treatments. **(C)** Comparison of the salt tolerance between 35S::*PtrTCP10* OE lines and wild type under salt treatments with different concentrations of NaCl. **(D,E)** Comparison of root length **(D)** and fresh weight **(E)** between 35S::*PtrTCP10* OE lines and wild type under salt treatments. Error bars indicate the SD. Asterisks represent a significant difference between 35S::*PtrTCP10* OE lines and wild-type (*p < 0.05, **p < 0.01; Student’s *t*-test).

To further investigate the role of *PtrTCP10* under cold stress, we generated 35S::*PtrTCP10* OE lines in *Arabidopsis*. The candidate positive seedlings screened on the hygromycin-resistant plates were verified by PCR and qRT-PCR (Supplementary Figure 2), and three independent 35S::*PtrTCP10* OE lines (i.e., OE1, OE3, and OE6) with high expression levels of *PtrTCP10* were selected for further study. Under normal condition, we found that the OE lines showed a similar growth as the wild-type (Col-0), and in contrast, under freezing condition, they exhibited freezing tolerance phenotypes with or without cold acclimation (Figure 8A). Statistically, their survival rates were significantly higher than the wild type (Figure 8B). Given that *PtrTCP10* could also be significantly induced by salt treatment at 7 days (Figures 4, 6), we examined the phenotypes of 35S::*PtrTCP10* OE lines under salt treatments. Compared with the wild type, the OE lines were more sensitive to salt stress (Figure 9). Their germination rate, root length, and fresh weight were significantly lower than the wild type. The findings suggested that *PtrTCP10* was positively involved in plant resistance to cold stress but also might be a negative regulator involved in salt stress. Then, we elucidated the regulatory molecular mechanism of *PtrTCP10* in response to cold and salt stresses.

### PtrTCP10-Mediated Regulatory Network Provides Molecular Mechanisms for Emerging Roles of *PtrTCP* Genes in Response to Cold/Salt Stresses

**FIGURE 10:**
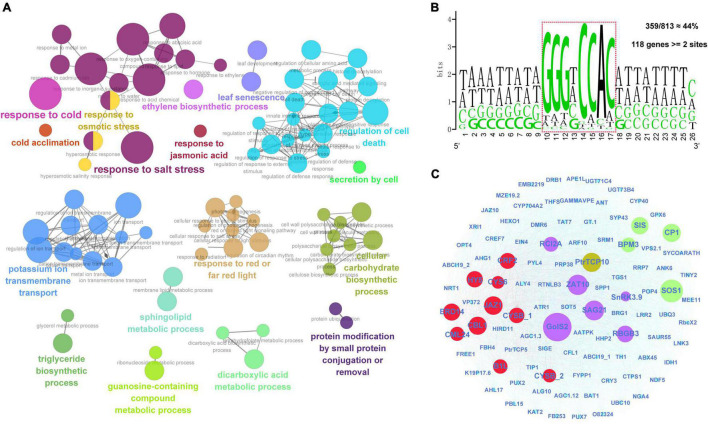
PtrTCP10-mediated regulatory network under cold and salt stresses. **(A)** Functionally grouped networks on the enriched GO terms from the co-expressed genes of *PtrTCP10* under cold and salt stresses. The enriched terms were linked according to their *K* score (≥0.3). **(B)** An enrichment of *PtrTCP10* binding cis-regulatory elements in promoters (1-kb) of the 813 co-expressed genes. The sequence in dotted box indicates the core region potentially bind by *PtrTCP10*. Approximately, 44% (359 out of 813) of the *PtrTCP10* co-expressed genes’ promoters contained this *cis*-regulatory element, and 118 *PtrTCP10* co-expressed genes contained at least two elements. **(C)** PtrTCP10-mediated regulatory network based on the high-confidence target genes of PtrTCP10. The red and green circles represent the well-known cold and salt responsive gene, and the purple circles represent the genes that could respond to both the cold and salt stresses.

First, we identified a total of 813 significantly co-expressed genes of *PtrTCP10* from the above transcriptome data under cold and salt stresses. The co-expressed genes were significantly enriched in the GO terms related to environmental stresses, such as response to cold, response to salt stress, response to osmotic stress, regulation of cell death, potassium ion transmembrane transport, and leaf senescence (Figure 10A). TCP transcription factors have been shown to recognize GC-rich *cis*-acting regulatory elements, and the class II TCP proteins show a preference binding for the motif GTGGNCCC (Kosugi and Ohashi, 2002; Viola et al., 2011; Manassero et al., 2013). To predict the putative target genes of PtrTCP10, we searched for the motif allowing a degenerate mutation against the promoters (1-kb upstream of TIS) of the 813 co-expressed genes. Approximately, 44% (359 out of 813) of the genes contained the motif, and of which, 118 genes contained at least two binding sites (Figure 10B). Based on the putative PtrTCP10 target genes (Supplementary Table 7), we constructed a regulatory network mediated by PtrTCP10. The network presented some well-acknowledged cold- and salt-responsive genes (e.g., *ZAT10*, *GolS2*, *HY5*, *CBL1*, *SOS1*, *RCI2A*, and *SnRK3.9*) directly regulated by PtrTCP10, which suggested that PtrTCP10 might be involved in response to cold and salt stresses by regulating those stress-related genes (Figure 10C; Wu et al., 1996; Capel et al., 1997; Guo et al., 2001; Cheong et al., 2003; Sakamoto et al., 2004; Nishizawa et al., 2008; Zhang et al., 2020).

## Discussion

Since the discovery of *TCP* genes, TCP transcription factors have been widely studied to be involved in multiple processes of plant growth and development, such as branching, floral organ morphogenesis, cell proliferation, leaf development, seed germination, regulation of circadian clock, and influencing of hormone pathways (Danisman et al., 2013). Recent studies suggested that they are also involved in environmental challenges, such as plant pathogen, high light stress, salt, and nutritional stress (Danisman et al., 2013; Guan et al., 2014; Mukhopadhyay and Tyagi, 2015; Danisman, 2016; Viola et al., 2016), but evidence for their innovation and evolutionary role in abiotic stress has been lacking. In this study, we identified *PtrTCP* family genes and traced their evolutionary trajectory. WGD events were the main underlying mechanism for expanding and innovating *PtrTCP* family genes. Comparative genomes showed 50% more *TCP* genes in *P. trichocarpa* than in *Arabidopsis*, and such significant innovation was produced by rWGD in *Populus* lineage after its divergence from *Arabidopsis.* Interestingly, the rWGD occurred around K-Pg extinction event ∼66 million years ago, which was followed by global cooling (Wu et al., 2020). Those rWGD-duplicated *PtTCP* genes might have undergone subfunctionalization and/or neofunctionalization during the severe cooling stress. Further expression profiles of *PtrTCP* genes from RNA-seq and qRT-PCR analysis showed at least one half can be differentially regulated by cold, heat, salt, and/or drought stresses. Moreover, the co-expression network identified a cold-associated regulatory module including *PtrTCP31, PtrTCP10*, and *PtrTCP36.* Of the three genes, *PtrTCP10* was rWGD-duplicated from *PtrTCP31*, evolved a strong capability of cold induction, and produced *PtrTCP36* with the capability of cold induction as well. In contrast, their co-orthologs in the other five dicots showed monotonous expression profiles after cold treatment. We questioned that the innovation of *PtrTCP10* and *PtrTCP36* with a strong capability of cold induction might contribute to the cold adaptation of the plants during global cooling.

Evidentially, OE of *PtrTCP10* in *Arabidopsis* increased freezing tolerance and salt sensitivity, indicating that *PtrTCP10* may be a regulator that plays a vital role in plants’ response to cold and salt stresses. Reconstruction of a PtrTCP10-mediated regulatory network indicated that PtrTCP10 might be involved in the regulation of plants response to cold and salt stresses by directly regulating the cold- and salt-relevant genes (e.g., *ZAT10*, *GolS2*, *HY5*, *CBL1*, *SOS1*, *RCI2A*, and *SnRK3.9*) (Wu et al., 1996; Capel et al., 1997; Guo et al., 2001; Cheong et al., 2003; Sakamoto et al., 2004; Nishizawa et al., 2008; Zhang et al., 2020). Overall, our work provides new insights into the innovation of *PtrTCP* genes and their emerging roles under abiotic stresses in woody plants, and meanwhile, it may provide valuable information for exploring the molecular mechanisms of *TCP* genes in improving plants resistance to abiotic stresses.

## Data Availability Statement

The datasets presented in this study can be found in online repositories. The names of the repository/repositories and accession number(s) can be found in the article/Supplementary Material.

## Author Contributions

SW, SL, and WW designed the research. SW, YS, and XZ performed the experiments. LG, LT, YN, and DD performed the bioinformatic analysis. YY and XY analyzed RNA-seq data. SW and WW wrote and revised the manuscript. All authors contributed to the article and approved the submitted version.

## Conflict of Interest

The authors declare that the research was conducted in the absence of any commercial or financial relationships that could be construed as a potential conflict of interest.

## Publisher’s Note

All claims expressed in this article are solely those of the authors and do not necessarily represent those of their affiliated organizations, or those of the publisher, the editors and the reviewers. Any product that may be evaluated in this article, or claim that may be made by its manufacturer, is not guaranteed or endorsed by the publisher.

## Footnotes

1
http://hmmer.org/
