# Supplementary material for: Innovation and Emerging Roles of Populus trichocarpa TEOSINTE BRANCHED1/CYCLOIDEA/PROLIFERATING CELL FACTOR Transcription Factors in Abiotic Stresses by Whole-Genome Duplication
Source: Front Plant Sci. 2022 Mar 9;13:850064. doi: 10.3389/fpls.2022.850064 (PMC8959825; doi:10.3389/fpls.2022.850064)
Supplement: Supplementary file 1 [file Data_Sheet_1.doc]

**Supplementary Figure 1**

**
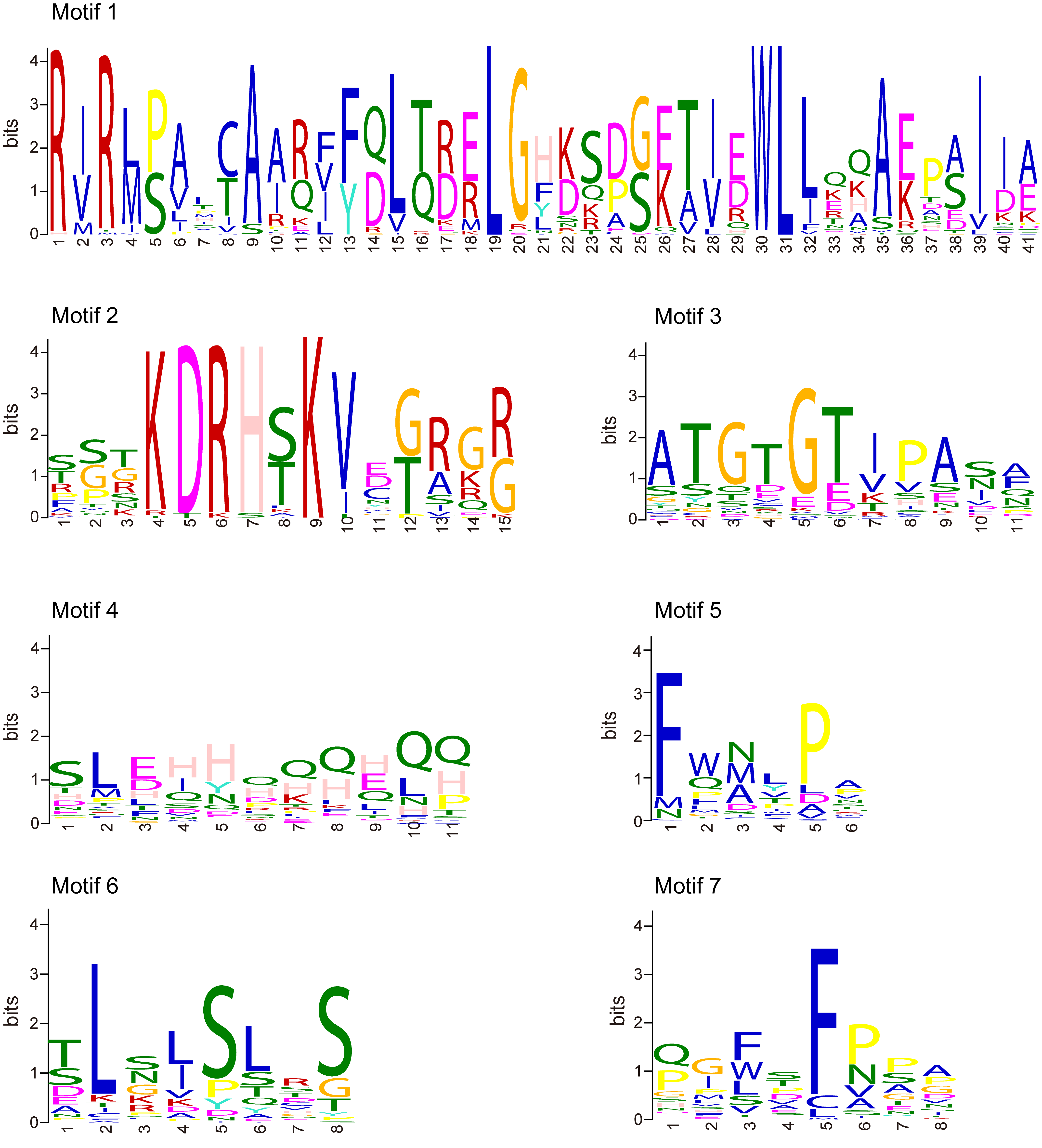
**

**Supplementary Figure 1.** The conserved motifs of PtrTCP proteins

**Supplementary Figure 2**

**
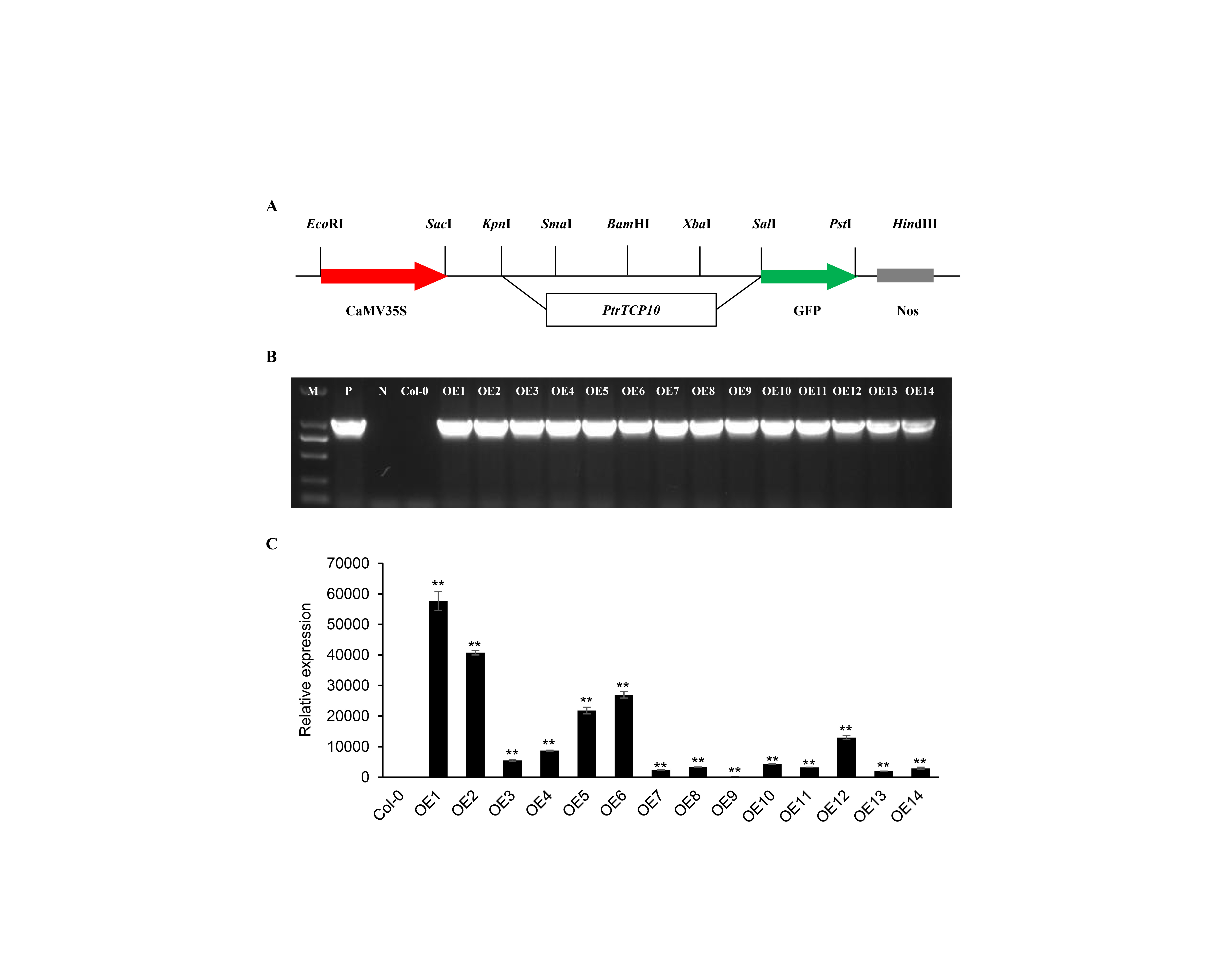
**

**Supplementary Figure 2. Generation of 35S::*PtrTCP10* overexpression lines in *Arabidopsis***

(A) Diagram of 35S::*PtrTCP10* overexpression vector (pCAMBIA1300-*PtrTCP10*-sGFP). The CDS sequence of *PtrTCP10* without termination codon was inserted into pCAMBIA1300-sGFP vector within *Kpn*I and *Sal*I sites. (B) PCR validation of the 35S::*PtrTCP10* overexpression lines (OE1-OE14). M, DNA Marker DL2000. 35S::*PtrTCP10* overexpression vector and deionized water were utilized as templates for the positive (P) and negative (N) controls. (C) Detection of the transcript levels of *PtrTCP10* in Col-0 and 35S::*PtrTCP10* overexpression lines by qRT-PCR, and the relative expression levels were normalized to *ACTIN2* (*AT3G18780*) and *ACTIN8* (*AT1G49240*). Each qRT-PCR experiment was performed three biological replicates and the error bar represents SD. Asterisks represent a significant difference between 35S::*PtrTCP10* overexpression lines and Col-0 (***p* < 0.01; Student’s *t*-test).
